# Elucidating the Interacting Domains of *Chandipura* Virus Nucleocapsid Protein

**DOI:** 10.1155/2013/594319

**Published:** 2013-10-28

**Authors:** Kapila Kumar, Sreejith Rajasekharan, Sahil Gulati, Jyoti Rana, Reema Gabrani, Chakresh K. Jain, Amita Gupta, Vijay K. Chaudhary, Sanjay Gupta

**Affiliations:** ^1^Center for Emerging Diseases, Department of Biotechnology, Jaypee Institute of Information Technology, A-10, Sector 62, Noida, Uttar Pradesh 201 307, India; ^2^Department of Microbiology, University of Delhi, Benito Juarez Marg, New Delhi 110021, India; ^3^Department of Biochemistry, University of Delhi, Benito Juarez Marg, New Delhi 110021, India

## Abstract

The nucleocapsid (N) protein of *Chandipura* virus (CHPV) plays a crucial role in viral life cycle, besides being an important structural component of the virion through proper organization of its interactions with other viral proteins. In a recent study, the authors had mapped the associations among CHPV proteins and shown that N protein interacts with four of the viral proteins: N, phosphoprotein (P), matrix protein (M), and glycoprotein (G). The present study aimed to distinguish the regions of CHPV N protein responsible for its interactions with other viral proteins. In this direction, we have generated the structure of CHPV N protein by homology modeling using SWISS-MODEL workspace and Accelrys Discovery Studio client 2.55 and mapped the domains of N protein using PiSQRD. The interactions of N protein fragments with other proteins were determined by ZDOCK rigid-body docking method and validated by yeast two-hybrid and ELISA. The study revealed a unique binding site, comprising of amino acids 1–30 at the N terminus of the nucleocapsid protein (N1) that is instrumental in its interactions with N, P, M, and G proteins. It was also observed that N2 associates with N and G proteins while N3 interacts with N, P, and M proteins.

## 1. Introduction

*Chandipura* virus (CHPV) is a recently recognized emerging human pathogen [1–3] of the genus *Vesiculovirus* and family Rhabdoviridae [4]. The ~11 kb genome of CHPV [5] is encapsidated by nucleocapsid (N) protein and serves as a template for both replication and transcription. The transcription of the genome by viral encoded RNA-dependent RNA polymerase (RdRp; L protein) produces five capped and polyadenylated mRNAs which code for five proteins nucleocapsid protein (N), phosphoprotein (P), matrix protein (M), glycoprotein (G), and large protein (L) in sequential order and in decreasing amounts [6]. Interactions among these proteins are essential for functioning of key processes during virus replication and pathogenesis. However, only few details of the molecular functions of these viral proteins that orchestrate the virus life cycle are known.

The N protein plays a pivotal role in virus biology by virtue of its interactions with other viral proteins. The interaction of monomeric N protein with P maintains it in the encapsidation competent soluble (active) form [7, 8]. In its active form, N protein tightly wraps the RNA genome and maintains the structural integrity along with the template function of the negative strand genome RNA. Within the virion, this encapsidated RNA (N-RNA) template is associated with the transcription factor (P protein) of the RNA polymerase complex to form the transcribing ribonucleoprotein (RNP) particle [9]. During transcription, the RNA polymerase complex (L and P) interacts with the N protein of the N-RNA template to transiently displace N and gain access to the genomic RNA [10]. The association of N (as part of the RNP particle) with M enables the condensation of the enwrapped genome thus giving the virion its characteristic bullet shape [11]. Earlier research had mapped certain interactions of CHPV such as NN and NP at the domain level [12, 13]. Deletion studies had shown that the N-terminal 47 amino acids (aa) together with residues 180–264 are indispensable for N protein oligomerization [12]. The N-terminal 180 aa and the C-terminal 102 aa of N protein have been mapped to be required for binding with P protein in its monomeric and RNA encapsidated state, respectively [13]. With the elucidation of interactions of N protein with M and G proteins [14], there arises a need to further map the specific regions of CHPV N protein involved in mediating its interactions.

In the current study, the structures of CHPV N and M proteins were generated by homology modeling using SWISS-MODEL workspace, G protein using I-TASSER, and P protein using a modified *abinitio* method. The nucleocapsid protein was divided into three fragments using PiSQRD—an N-terminal region N1 (Nt arm), a central region N2 (Nt lobe), and a C-terminal region N3 (Ct lobe). The involvement of N protein fragments in interactions of N with N, P, M, and G proteins was predicted using ZDOCK rigid-body docking method and further checked and validated by yeast two-hybrid system and ELISA-based assay.

## 2. Materials and Methods

### 2.1. Structure Elucidation of Chandipura Virus Proteins by Homology Modeling

The structural models of N and M proteins of CHPV were generated by SWISS-MODEL workspace. Vesicular stomatitis virus (VSV), Indiana strain N protein complex (PDB ID: 3HHW), and M protein (PDB ID: 1LG7) were used as templates for homology modeling of CHPV proteins as both these viruses belong to the same genus (genus *Vesiculovirus*) and their N and M proteins shares 71.3% and 28% sequence similarity, respectively. The structure of CHPV G protein in its perfusion state (530 aa) was generated using I-TASSER [15, 16] and the atomic clashes along with bond length errors within the structure were removed by using ModRefiner [17]. Threading-based modeling was used for G protein since its full-length template (VSV G) was not available in PDB, unlike N and M proteins, whose structures were available for VSV. Modeling of CHPV P protein structure using homology modeling and threading was not feasible due to the unavailability of full-length structural homologs. VSV P protein oligomerization domain (PDB ID: 2FQM) and C-terminal domain (PDB ID: 2 K47) were the only available templates that exhibited homology to the corresponding domains of CHPV P protein. The structure of CHPV P protein was hence modeled using *abinitio* method in combination with protein folding constraints. CHPV P protein was divided into four segments: N-terminal domain (residues 1–103), oligomerization domain (residues 104–168), interconnecting domain (residues 169–215), and C-terminal domain (residues 216–289). Oligomerization and C-terminal domains were modeled using SWISS-MODEL workspace while N-terminal and interconnecting domain models were obtained using I-TASSER and QUARK [18], respectively. The disulfide connectivity was predicted using DiANNA server [19]. The complete model was assembled manually by reproducing the torsion angles from the individual modeled segments to the extended polypeptide chain (289 amino acid) using Swiss-PDB Viewer. The torsion angles were changed wherever necessary based on disulfide connectivity and secondary structure prediction. CHPV protein structures thus generated were subjected to prepare protein protocol of Accelrys Discovery Studio Client 2.55 for the energy minimization, optimizing short/medium sized loop regions, and protonating the protein structures. The stereochemical quality was estimated using Ramachandran plot, Verify3D, and ERRAT.

### 2.2. Decomposing N Protein into Quasi-Rigid Dynamic Fragments

PiSQRD web resource was used to subdivide CHPV N protein structure in quasi-rigid fragments. This server uses an algorithm introduced by Potestio and coworkers [20] and subdivides proteins into regions that behave approximately as rigid units in the course of protein structural fluctuations. Default values were used for all the parameters with captured mobility threshold of 80% and 10 lowest energy essential modes.

### 2.3. Docking of CHPV N Protein

Docking of CHPV N protein with N, P, M, and G proteins was performed using the ZDOCK (Accelrys) [21, 22] rigid-body docking method. ZDOCK provides rigid body docking of two protein structures using the ZDOCK algorithm as well as clustering the poses according to the ligand position. RDOCK (Accelrys) [23, 24] refinement was performed on the top 100 poses of the filtered ZDOCK output of each interacting pair. A distance, dependent dielectric constant 4*r* (*r* being the distance) was used during refinement.

### 2.4. Construction of Yeast Two-Hybrid Plasmids

The putative nucleocapsid (N) fragments (N1, N2, and N3) were amplified using specific primers (as listed in Table 1) designed to incorporate *Nde*I and *Bam*HI restriction enzyme sequences at their 5′ ends to facilitate cloning in yeast expression vectors pGBKT7 (BD, bait) and pGADT7 (AD, prey), Clontech, USA. These primer pairs span nucleotides 1–90 bp (N1), 61–894 bp (N2), and 693–1260 bp (N3) of the complete nucleocapsid ORF cloned in pET33b vector [7]. The clone of CHPV N gene was a kind gift from Dr. Dhrubajyoti Chattopadhyay of Dr. B. C. Guha Centre for Genetic Engineering, Kolkata, India.

The N protein fragments were amplified by standard polymerase chain reaction (PCR) as described previously [14], purified using PCR clean up kit (Sigma Aldrich, USA), and digested with *Nde*I and *Bam*HI restriction enzymes(Fermentas, USA). The vectors, pGBKT7 and pGADT7, were linearized by the same enzyme combination and subsequently ligated with the digested N protein fragments using T4 DNA Ligase (5 U/*μ*L, Fermentas, USA) to generate the respective bait and prey constructs. The resulting pGBKT7 constructs, BD-N1, BD-N2, and BD-N3, encoded the fragments N1 (30 amino acids), N2 (278 amino acids), and N3 (191 amino acids) fused in frame at the C terminus of BD domain and the pGADT7 constructs, AD-N1, AD-N2, and AD-N3, encoded the corresponding fragments fused in frame downstream of AD domain. The complete ORFs encoding N, P, M, and G proteins of CHPV as both BD and AD fusions (BD-N, BD-P, BD-M, BD-G, AD-N, AD-P, AD-M, and AD-G) used in this study have been described earlier by the authors [14].

### 2.5. Construction of Bacterial Expression Plasmids

Deletion constructs for ELISA were generated by PCR amplification using primers corresponding to the appropriate end sequences with added *Bam*HI (5′) and *Xho*I (3′) sites (Table 1) and N-pET33b as template [7]. The amplified products were digested with *Bam*HI and *Xho*I, purified, and subcloned into pGEX-4T3 vector (GST tag) digested with the same enzyme combination. The recombinants were confirmed by restriction enzyme digestion. The pGEX-4T3 constructs called GST-N1, GST-N2, and GST-N3 contain the three fragments fused in frame with GST tag at the N terminus.

### 2.6. Yeast Transformation

*Saccharomyces cerevisiae* strains Y187 and AH109 were used for protein interaction analysis. These strains were transformed individually with BD and AD constructs, respectively, following lithium acetate yeast transformation protocol as explained by manufacturer (Matchmaker GAL4 two-hybrid system 3 and libraries user manual, protocol number PT3247-1). Successful transformants were screened on Synthetically Defined (SD, Clontech, USA) media lacking amino acids tryptophan and leucine (selection marker for BD and AD plasmids, resp.). The constructs were also screened for autologous activation of the reporter gene *HIS3* on SD media lacking tryptophan and histidine (SD/-Trp/-His) for bait constructs and on SD media lacking leucine and histidine (SD/-Leu/-His) for prey constructs.

### 2.7. Yeast Two-Hybrid Screening

Each of the bait construct in Y187 yeast strain was allowed to mate with each prey construct in AH109 yeast strain. All three fragments in BD/AD (N1, N2, and N3) vector were mated with four complete ORFs (N, P, M, and G) in AD/BD vector accounting for a total of 24 mated combinations. Successfully mated diploids containing both bait and prey vectors were selected on SD media lacking tryptophan and leucine (SD/-Trp/-Leu) and tested for positive protein interaction by plating on SD/-Trp/-Leu/-His media. Simultaneously, the mated clones were screened on SD/-Trp/-Leu/-His/*α*-gal plate for *α*-galactosidase assay. The development of blue color in the presence of X-*α*-gal was indicative of positive interaction in this assay. 

### 2.8. Enzyme Linked Immunosorbent Assay (ELISA) for Interaction Validation

ELISA was performed as a second independent method to check the interactions of N proteins fragments with N, P, M, and G proteins. Streptactin-coated microtiter plate (IBA-GmBH, Germany) was used to check the interactions between full-length CHPV proteins as Strep tag fusions (Strep-N, Strep-P, Strep-M, and Strep-G), generated by the authors in previous study [14], and nucleocapsid protein fragments as GST fusions (GST-N1, GST-N2, and GST-N3). The protocol involved has been described earlier by the authors [14, 25].

## 3. Results

### 3.1. Model Building and Validation

CHPV P protein structure was transformed locally to introduce disulfide links between Cys37–Cys172 and Cys57–Cys286 in concordance with the predicted disulfide linkages. Ramachandran plot analysis displayed approximately 98% of the residues in allowed region (favored + allowed regions) and the rest were outliers. Approximately 99.3% residues of CHPV N protein lie in the allowed regions. Verify 3D and ERRAT analysis indicated good quality of all the protein models with minimal interatomic clashes. 

### 3.2. Rigid-Body Docking for Domain-Protein Assembly

**Figure 1:**
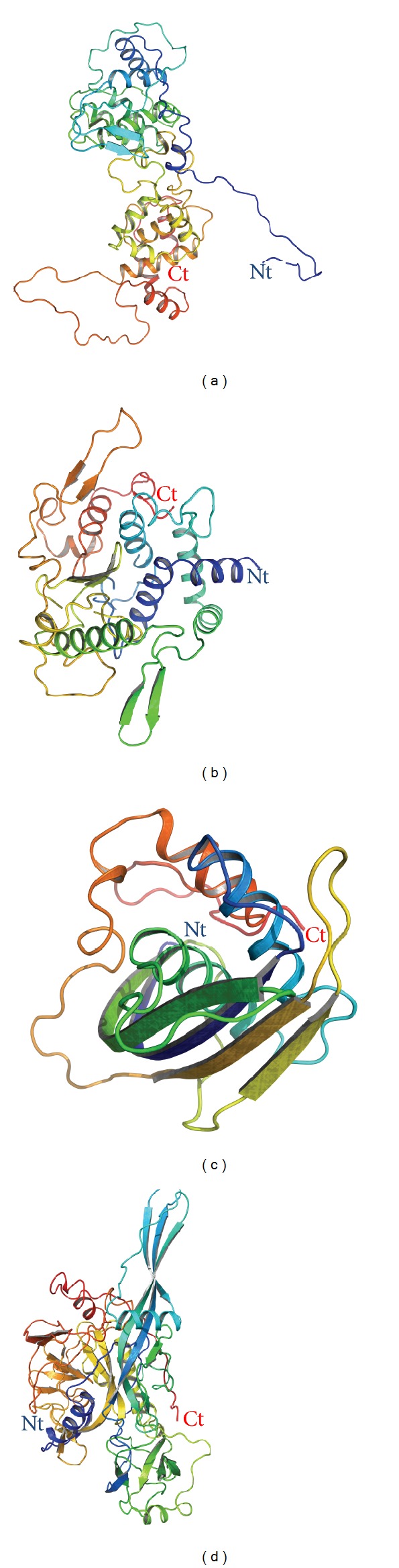
Structures of N, P, M, and G proteins of CHPV predicted using SWISS-MODEL workspace. (a) Nucleocapsid protein, (b) phosphoprotein, (c) matrix protein, and (d) glycoprotein of Chandipura virus rendered in cartoon (rainbow color).

**Figure 2:**
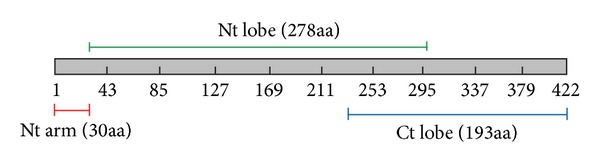
Schematic representation of the CHPV nucleocapsid (N) protein fragments. N terminal fragment 1 (N1) is of 30 amino acids (aa). Fragment 2 (N2) is of 278 aa. The N terminal 10 aa residues and the C terminal 68 aa residues of N2 overlaps with N1 and N3, respectively. Fragment 3 (N3) constitutes the C terminal 193 aa of the CHPV N protein.

**Figure 3:**
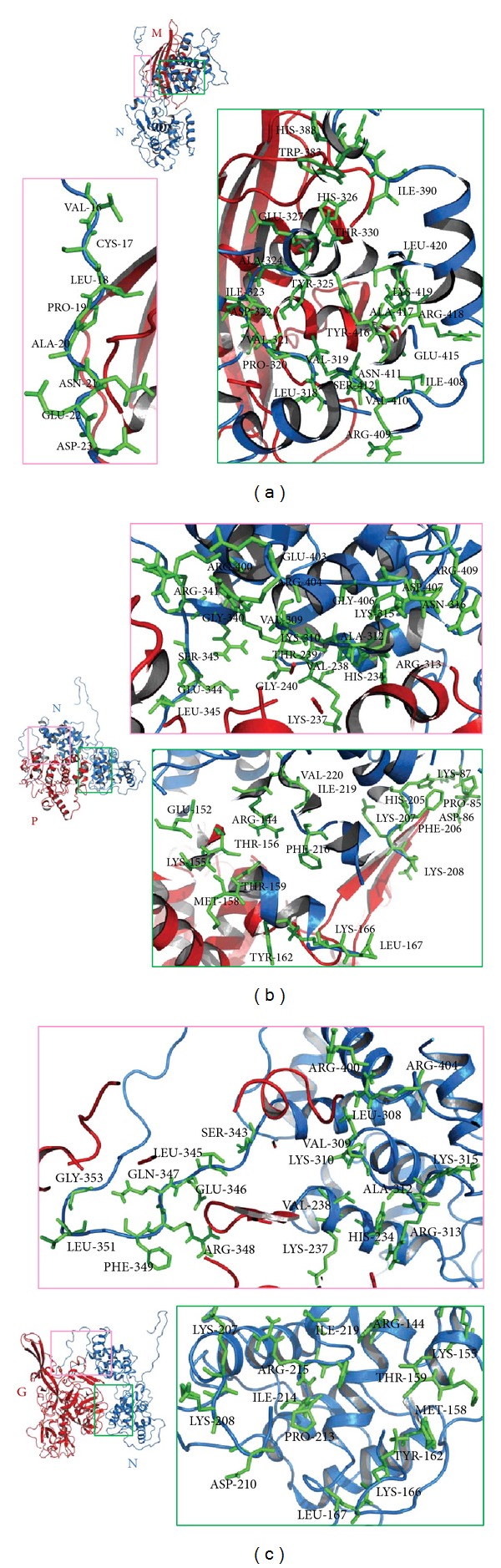
Docking results for NM, NP, and NG interactions using ZDOCK. (a) Top rank NM complex ZDOCK pose 684 (E_RDOCK score—31.356). (b) Top rank NP complex ZDOCK pose 220 (E_RDOCK score—27.932). (c) Top rank NG complex ZDOCK pose 8 (E_RDOCK score—40.982). CHPV proteins are represented as cartoons models. CHPV N protein is shown in blue and M, P, and G proteins are shown in red. (a) The residues of CHPV N Protein in the NM interface correspond to N1 and N2. (b) The residues of CHPV N Protein in the NP interface correspond to N1 and N3. (c) The residues of CHPV N protein in the NG interface correspond to N2 and N3 fragments. The NN interaction has been discussed previously by the authors [26].

CHPV full-length protein structures (N (Figure 1(a)), P (Figure 1(b)), M (Figure 1(c)), and G (Figure 1(d))) were docked on CHPV N protein model as rigid-bodies using ZDOCK at a 15° rotational sampling density. Top 2000 poses were further reranked (ZRank) using detailed electrostatics, van der Waals, and desolvation energy terms. The success of the resulting predictions was evaluated based on their RMSD values. An acceptable docking pose has been defined as one where the RMSD of one of the proteins is ≤10 Å from the cluster center (the choice of 10 Å RMSD to define an acceptable pose is in concordance with the ligand RMSD used by CAPRI to define an acceptable solution in protein-protein docking). The best pose with minimum E RDOCK score, generated after RDOCK refinement, was chosen as near-native structure for each interaction. It was observed that N terminal region of N protein (N1, Nt arm) interacts with N, P, and M proteins while the central N2 (Nt lobe) interacts with N, P, and G proteins and C terminal region N3 (Ct lobe) interacts with N, P, M, and G proteins (Figures 2 and 3). These putative interactions were experimentally checked by Y2H and ELISA methods.

### 3.3. Screening for Potential Positive Interactions by Y2H Analysis

The Y2H bait (pGBKT7) and prey (pGADT7) recombinants containing N1, N2, and N3 were transformed in haploid *S. cerevisiae* strains Y187 and AH109, respectively. The positive transformants were selected on SD/-Trp media for bait fusion and SD/-Leu media for prey fusion vectors. Prior to the Y2H interaction analysis, generated bait fusion constructs were checked for their ability to activate the expression of the *HIS3 *reporter gene (autoactivation) on histidine-deficient SD media (SD/-Trp/-His for bait constructs and SD/-Leu/-His for prey constructs). None of the hybrid bait or prey vectors tested in the study showed background transcriptional activity (data not shown) and thus were found to be suitable for Y2H studies. However, N1 and N2 fragments as AD fusions were observed to interact with empty BD vector (control vector) to activate the reporter genes *HIS3* and *MEL1 *(Figure 5, sectors 28 and 29). Thus, the interactions involving N1 and N2 fragments were analysed using BD-N1 and BD-N2. Full-length CHPV viral genes had been previously transformed in corresponding yeast strains and checked for autoactivation in earlier studies by the authors [14]. BD-P was found to activate the histidine reporter gene and thus combinations involving P protein were studied in context of reverse direction taking P protein as AD fusion.

**Figure 4:**
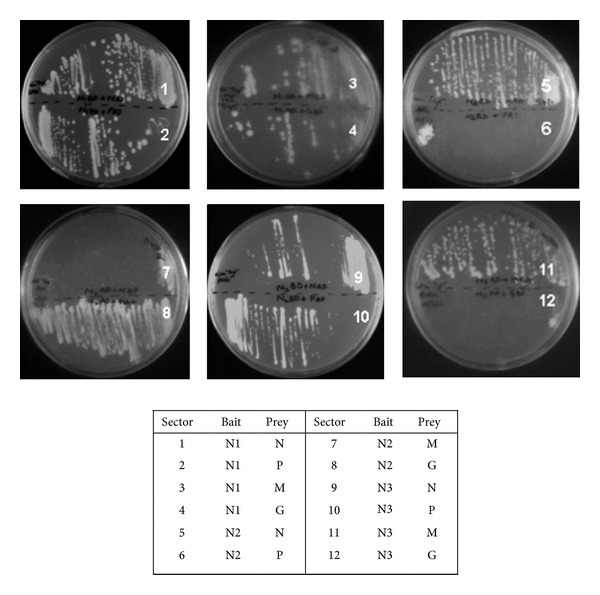
Screening of CHPV N1, N2, and N3 interactions with N, P, M, and G proteins by Y2H. All four viral genes cloned individually as DNA-binding domain fusion (bait) and transformed in Y187 yeast strain were systematically mated with N1, N2, and N3 cloned individually as DNA-activation domain fusions (prey), transformed in AH109 yeast strain. The diploid cells formed were selected on SD medium lacking amino acids tryptophan and leucine. Putative positive interactions were tested on SD media lacking tryptophan, leucine, and histidine All combinations were tested in at least 2 independent experiments. Presence of growth on medium (sectors 1, 2, 3, 4, 5, 8, 9, 10, and 11) indicated interaction between the proteins while absence (sectors 6, 7, and 12) suggested no interaction.

**Figure 5:**
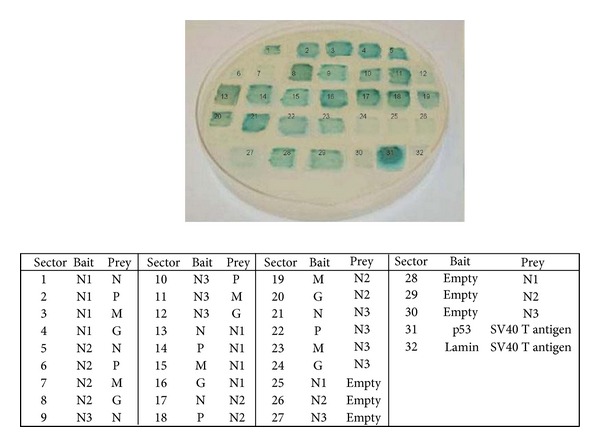
X-Alpha galactosidase assay for interaction confirmation. All possible interacting pairs of CHPV nucleocapsid protein with other viral proteins with controls were plated in an array format on plate containing X-*α*-gal (SD/-Trp/-Leu/-His/*α*-gal). Interactions were considered in both directions, that is, as BD and AD fusions. The results obtained were consistent in both directions except for BD-P which was autoactivating (sectors; 14, 18, and 22). Production of blue color reconfirmed the positive interactions observed in Y2H analysis while absence of color and growth is an indication of a noninteracting protein pair.

Yeast strains carrying bait and prey constructs were mated and the resulting diploids were screened under selective conditions on SD/-Trp/-Leu media. Each combination of proteins was considered in both directions, that is, as both BD as well as AD fusions to test for the interactions of N1, N2, and N3 with full-length N, P, M, and G proteins (Table 2). Mated diploids were checked for potential positive interaction by analyzing expression of reporter genes* HIS3* and *MEL1*. The expression of *HIS3* was analysed by growth on SD media lacking tryptophan, leucine, and histidine (SD/-Trp/-Leu/-His). The colonies which grew on histidine-deficient media were considered to be positive (Figure 4). Activation of another reporter gene, that is, *MEL1*, was also checked by plating on SD/-Trp/-Leu/-His/*α*-gal media and blue colored colonies indicated positive interactions (Figure 5). Tumor suppressor protein p53 and Simian virus large T-antigen encoded by pGBKT7-53 and pGADT7-T vectors as BD and AD fusions are known to be interacting proteins and thus were taken as positive control (Figure 5, sector 31), whereas pGBKT7-Lam and pGADT7-T encoding noninteracting Lamin protein and Simian virus large T-antigen served as negative interaction control (Figure 5, sector 32). It was observed that 8 (interaction of N1 with N, P, and M proteins; N2 with N and G proteins and N3 with N, P, and M proteins) out of the 10 putative positive interactions identified through computational approach were positive in Y2H screening. In addition to the 8 associations, interaction of N1 fragment and G protein was also observed to be positive in Y2H.

### 3.4. Validation of Protein Interactions by ELISA Assay

**Figure 6:**
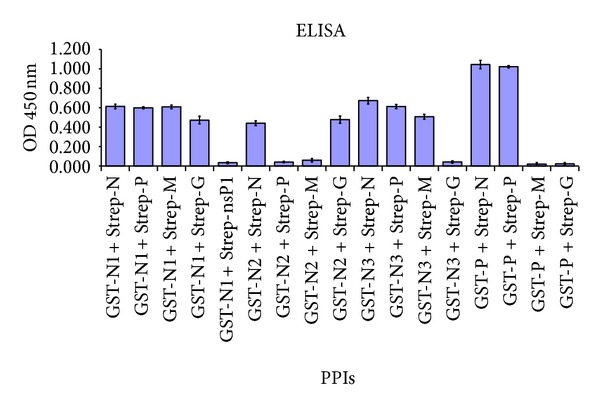
Validation of N protein fragment interactions with CHPV full-length proteins by ELISA. ELISA revealing the interaction between N1, N2, and N3 and full-length CHPV N, P, M, and G proteins. Only Strep tag fusion proteins (Strep-N, Strep-P, Strep-M, Strep-G, and Strep-nsP1) and only GST tag fusion proteins (GST-N1, GST-N2, GST-N3, and GST-P) were considered as background controls. A nonrelevant Strep tag fusion protein (Strep-nsP1) of Chikungunya virus was taken to test the specificity of N1 interactions. The data shown is mean of three independent experiments (mean ± SD). The known interaction pairs (PN and PP) and noninteractors (PM and PG) of CHPV were included as experimental controls. The absorbance at 450 nm is plotted on the *y* axis (10 division = 0.2 OD) and the test protein pairs are considered on the *x* axis.

Each pairwise combination tested by Y2H analysis was checked independently by ELISA assay to add to the reliability of the data obtained. The authors have previously used ELISA as a method of choice for identification of protein interactions [14, 25]. The truncated fragments of N protein cloned in pGEX-4T3 vector allowed for the expression of each fragment with a GST tag at the N terminus. Full-length CHPV N, P, M, and G proteins as Strep tag fusions were expressed and the cell lysates were prepared as described previously [14]. GST-N1, GST-N2, and GST-N3 were also expressed and the cell lysates were analysed for the solubility of these fragments. All three fragments were observed to be soluble (data not shown) and were used for the binding assay. Known interactions among full-length CHPV proteins interaction between P and N proteins involving GST-P+Strep-N and PP involving Strep-P+GST-P as positive controls, while non-interacting pairs PM as GST-P+Strep-M and PG involving GST-P+Strep-G as negative controls; 14 were taken as controls. The binding of N1 region with a nonrelevant protein-nonstructural protein 1 (nsP1) of Chikungunya virus (CHIKV) as Strep tag fusion [25] and the binding of Strep (Strep-N, Strep-P, Strep-M and Strep-G) and GST (GST-N1, GST-N2, and GST-N3) fusions directly with the Streptactin plate were also taken as experimental controls. Anti-GST antibody (primary antibody) followed by HRP-conjugated secondary antibody was used to check the protein interactions. The absorbance was measured at 450 nm after stopping the reaction of HRP with the substrate TMB using 2N HNO_3_. The experiments were performed in triplicates and their mean ± SD have been graphically represented in Figure 6.

Analysis of 12 pairs of putative protein interactions among N protein fragments and CHPV proteins revealed a total of 9 positive interactions. N1 (Nt arm) has been shown to interact with all four viral proteins, that is, N, P, M, and G. N2 (Nt lobe) was observed to associate with N and G protein while N3 (Ct lobe) bound to N, P, and M proteins (Table 2). The interactions observed in ELISA were in concordance with both Y2H analysis and the data available from the literature.

## 4. Discussion

During the life cycle of viruses, the encoded proteins extensively interact with one another to perform their functions. These protein-protein interactions (PPIs) are achieved through specific regions that are responsible for the physical interactions. These regions which mediate different interactions are considered as building blocks of interaction networks. Domain mapping has been carried out for several viruses such as Herpes Simplex Virus type I (HSV), Epstein Barr Virus (EBV), Kaposi's Sarcoma associated Human Virus (KSHV) [27], Murine Coronavirus [28], Rabies Virus (RV) [29], VSV [8, 30], and Sendai virus [31] and for certain proteins of CHPV as well [12, 13]. These studies have highlighted the importance of identifying the regions which can be targeted for therapeutic strategies.

In a recent study on intraviral protein interactions among Chandipura viral proteins, the authors had reported the interaction of N protein with N, P, M, and G proteins of CHPV [14]. In order to investigate the interacting regions of N protein, a series of interaction studies employing bioinformatics-based docking studies, yeast two-hybrid system, and ELISA have been performed. The division of N protein into putative fragments was guided in large part by our prediction of the protein structure. These predictions provide insights into the less known structures of CHPV proteins.

Each combination of nucleocapsid protein fragments and CHPV full-length proteins was tested using ZDOCK, Y2H, and ELISA, allowing us to assess the reliability of the data for protein interactions obtained in this study. The study identified the interacting residues involved in NN association which are present in all the three regions of nucleocapsid protein considered in this study (N1, N2, and N3). However, the central 278 aa region (N2) essential for interaction with G protein is shown to be dispensable for interactions with M and P proteins. The interaction dataset generated by Y2H and ELISA correlates with 75% of the ZDOCK based predictions and 100% with the data known from literature (Table 2).

Earlier mapping studies of CHPV N protein involved the generation of N protein fragments by enzymatic digestion using chymotrypsin [12, 13]. Nevertheless, our choice of boundaries for putative nucleocapsid protein fragments was based on precise structural and biophysical criteria. The interacting residues involved in NN association (previously reported by the authors; 31) lie within the N terminal and central regions of the N monomer as shown by Mondal and coworkers [12]. However, our bioinformatics predictions have narrowed down these regions to smaller peptides including residues 8 to 22 at the N terminus and 245 to 256 in the central region. Moreover, with evidence from the oligomerisation studies of CHPV N [26] and VSV N protein [30], we suggest the involvement of intermittent residues from 321 to 395 at the C terminus in the oligomerisation of N protein. Deletion studies in CHPV had shown the involvement of N terminal aa 1 to 180 and C terminal aa 320 to 390 of N protein in NP interaction. The present work after considering the N protein oligomerisation as well as RNA binding constraints suggests smaller peptides within these regions—residues 2 to 30, 140 to 165, 205 to 240, and 320 to 343, to be indispensable for NP association. In addition to identifying the interacting residues of N involved in NN and NP associations, we have also predicted the regions of N protein responsible for NM and NG interactions. The NM interaction involves the aa residues 16 to 20 and 318 to 420, while NG binding requires aa 144 to 240 in the central region of the N protein. Our data corroborates well with the previously identified interacting regions involved in NN and NP interactions for both CHPV [12, 13] and VSV [30], thus validating our approach of interaction analysis.

Although important data has been generated by mapping studies, the biological significance of these interactions is the scope of further experimentation. Nevertheless, these associations can prove to be valuable starting points for understanding CHPV biology and designing antiviral strategies. Components blocking the N protein interacting regions may represent a novel class of molecules suitable for a therapeutic intervention in Chandipura-mediated disease.

## Figures and Tables

**Table 1:** Primers used for cloning N gene domains of CHPV.

S. no.	Construct	Oligo name	Primer sequence
1	BD-N1 and AD-N1	N1 F *Nde*I (BD/AD)	GGAAGTGA**CATATG**AGTTCTCAAGTATTCTGC
		N1 R *Bam*HI (BD/AD)	GCTAACA**GGATCC**GAAGAATGCCCCTGGAAAC
2	BD-N2 and AD-N2	N2 F* Nde*I (BD/AD)	GGAAGTGA**CATATG**GAAGACCCAGTGGAGTTTC
		N2 R *Bam*HI (BD/AD)	GCTAACA**GGATCC**ATGGAAACTGGGATTTTTTGTTG
3	BD-N3 and AD-N3	N3 F* Nde*I (BD/AD)	GGAAGTGA**CATATG**ACTCTGTCACACCTCCAG
		N3 R* Bam*HI (BD/AD)	GCTAACA**GGATCC**TCATGCAAAGAGTTTCCTGGC
4	GST-N1	N1 F *Bam*HI (pGEX-4T3)	GCTAACA**GGATCC**ATGAGTTCTCAAGTATTCTGC
		N1 R *Xho*I (pGEX-4T3)	GCTAACA**CTCGAG**GAAGAATGCCCCTGGAAAC
5	GST-N2	N2 F *Bam*HI (pGEX-4T3)	GCTAACA**GGATCC**ATGGAAGACCCAGTGGAGTTTC
		N2 R *Xho*I (pGEX-4T3)	GCTAACA**CTCGAG**ATGGAAACTGGGATTTTTTGTTG
6	GST-N3	N3 F *Bam*HI (pGEX-4T3)	GCTAACA**GGATCC**ATGACTCTGTCACACCTCCAG
		N3 R *Xho*I (pGEX-4T3)	GCTAACA**CTCGAG**TCATGCAAAGAGTTTCCTGGC

Primers used for PCR amplification of Chandipura Virus N1, N2, and N3 domains of nucleocapsid gene (F: forward primer and R: reverse primer). The names of the restriction enzymes are in italics and their recognition sequences in bold.

**Table 2:** Cumulative results of CHPV N protein fragments interaction analysis.

Protein pair used for interaction analysis	Interaction analysis by ZDOCK and RDOCK	Y2H assay		ELISA	Known from the Literature
		*HIS3 *reporter (nutritional selection)	*MEL1 *reporter (*α*-galactosidase assay)		
N1-N	*√*	*√*	*√*	*√*	*√* [12]
N1-P	*√*	*√*	*√*	*√*	*√* [13]
N1-M	*√*	*√*	*√*	*√*	—
N1-G	X	*√*	*√*	*√*	—
N2-N	*√*	*√*	*√*	*√*	*√* [12]
N2-P	*√*	X	X	X	
N2-M	X	X	X	X	—
N2-G	*√*	*√*	*√*	*√*	—
N3-N	*√*	*√*	*√*	*√*	*√* [12]
N3-P	*√*	*√*	*√*	*√*	*√* [13]
N3-M	*√*	*√*	*√*	*√*	—
N3-G	*√*	X	X	X	—

*√* represents positive interaction.

X represents negative interaction.
